# Medical and Surgical Reporter

**Published:** 1858-05

**Authors:** S. W. Butler


### Med. & Surg. Reporter.

From the card below, it will be perceived, that the Medical and Surgical Reporter has been removed to Philadelphia.

It is one of our very best exchanges, and in seeking a wider field, we shall expect for it an enlarged success:

"BURLINGTON, N. J., April 1, 1858.

From this date the MEDICAL AND SURGICAL REPORTER will be issued *from Philadelphia*, and *Wm. B. Atkinson, M. D.*, of that city, will be associated with the undersigned in the editorial management of the work.

The *publishing department* will be in the hands of Mr. *J. W. Bradley*, 48 *North Fourth St. Philadelphia.*

All communications, books, &c., should be directed to the editors, care of Mr. Bradley.

☞ *Exchanges* will confer a favor by announcing the above change.

S. W. BUTLER, M. D."

